# Effective Deep Brain Stimulation for Obsessive-Compulsive Disorder Requires Clinical Expertise

**DOI:** 10.3389/fpsyg.2019.02294

**Published:** 2019-10-22

**Authors:** Maarten van Westen, Erik Rietveld, Damiaan Denys

**Affiliations:** ^1^Department of Psychiatry, Amsterdam UMC, University of Amsterdam, Amsterdam, Netherlands; ^2^Amsterdam Brain and Cognition, University of Amsterdam, Amsterdam, Netherlands; ^3^Institute for Logic, Language and Computation, University of Amsterdam, Amsterdam, Netherlands; ^4^Department of Philosophy, University of Twente, Enschede, Netherlands; ^5^Netherlands Institute for Neurosciences, Institute of the Royal Dutch Academy of Arts and Sciences, Amsterdam, Netherlands

**Keywords:** obsessive-compulsive disorder, deep brain stimulation, evaluation, decision-making, clinical expertise, radical embodied cognitive science, affordances

## Abstract

**Background:**

Deep brain stimulation (DBS) is an innovative treatment for severe obsessive-compulsive disorder (OCD). Electrodes implanted in specific brain areas allow clinicians to directly modulate neural activity. DBS affects symptomatology in a completely different way than established forms of treatment for OCD, such as psychotherapy or medication.

**Objective:**

To understand the process of improvement with DBS in patients with severe OCD.

**Methods:**

By means of open-ended interviews and participant observation we explore how expert clinicians involved in the post-operative process of DBS optimization evaluate DBS effects.

**Results:**

Evaluating DBS effect is an interactive and context-sensitive process that gradually unfolds over time and requires integration of different sources of knowledge. Clinicians direct DBS optimization toward a critical point where they sense that patients are *being moved* with regard to behavior, emotion, and active engagement, opening up possibilities for additional cognitive behavioral therapy (CBT).

**Discussion:**

Based on the theoretical framework of radical embodied cognitive science (RECS), we assume that clinical expertise manifests itself in the pattern of interaction between patient and clinician. To the expert clinician, this pattern reflects the patient’s openness to possibilities for action (“affordances”) offered by their environment. OCD patients’ improvement with DBS can be understood as a change in openness to their environment. The threshold for patients to engage in activities is decreased and a broader range of daily life and therapeutic activities becomes attractive. Movement is improvement.

## Introduction

Deep brain stimulation (DBS) is an innovative treatment in psychiatry. Electrodes implanted in specific brain areas allow clinicians to directly modulate neural activity. DBS is effective in reducing symptoms of patients with therapy-refractory obsessive-compulsive disorder (OCD) (Alonso et al., 2015). Since its introduction in 1999, 200-300 OCD patients have been treated worldwide. In contrast to established forms of treatment for OCD, such as psychotherapy or pharmacology, DBS affects symptomatology in a completely different way. DBS effects can occur very rapidly, sometimes in a matter of seconds (de Koning et al., 2016). Various OCD symptoms change on different time courses, with mood frequently improving almost instantaneously, obsessions over the course of weeks, and compulsions taking months to improve. It is unclear whether acute effects are predictive for long term improvement. Furthermore, DBS induces a broad range of changes in the phenomenology of patients, many of which are not captured by standardized clinical rating scales, such as the Yale-Brown Obsessive-Compulsive Scale (YBOCS) (de Haan et al., 2013, 2015, 2017). Moreover, patients themselves are initially often not aware of rather pronounced DBS-induced changes in their symptoms. This makes the effects of DBS difficult to interpret and to manage. The objective of this study is, therefore, to explore the process of improvement with DBS in patients with severe OCD.

## Materials and Methods

### Setting

This study was conducted at the Psychiatry department of the Amsterdam University Medical Centre, location AMC. Since 2005 these clinicians have been treating over 80 therapy-refractory OCD patients with DBS in the ventral anterior limb of the internal capsule (vALIC). Their clinical expertise offers a unique perspective on the effects of DBS on patients. In this study, we try to understand the effects of DBS from the perspective of expert clinicians involved in the process of DBS optimization.

Deep brain stimulation optimization is a post-operative treatment phase in which the electrical stimulation is optimized with regard to symptom reduction and side effect suppression (Morishita et al., 2014). DBS optimization is an iterative process in which evaluation of DBS effects takes a central role. First, clinicians adjust stimulation parameters, like voltage, frequency, contact configuration, and pulse width. Second, they evaluate the effects of each such adjustment on OCD symptoms. Based on this evaluation they make further adjustments in stimulation parameters, and so on and so forth.

### Interviews

All clinicians involved in DBS stimulation parameter optimization, which were four psychiatrists, two psychologists, and two specialized nurses, were interviewed for 2 h between July and November 2016. The interviews were open-ended, thereby minimally distorting with pre-defined categories the clinicians’ descriptions of their own ideas and experiences. Two main questions were posed: “How do you characterize an optimal DBS effect?” and “What do you do to evaluate DBS effect?”. Furthermore, we made use of a flexible topic list, which we adjusted when new topics emerged. When clinicians did not come up with a certain topic on our list, we addressed it ourselves at the end of the interview. Interviews were recorded and transcribed verbatim. Clinicians read their own and each other’s transcripts and commented on it in a focus group.

### Observations

The first author was embedded in the team and participated in the treatment process to the extent that he interacted with clinicians and patients, without performing any therapeutic actions *per se*. Clinicians were observed at various occasions: during their sessions with patients when stimulation parameters were adjusted, during weekly meetings of the multidisciplinary treatment team, training sessions for new DBS clinicians, brainstorm sessions on new strategies of optimizing stimulation parameters, and sessions of additional therapy, such as cognitive behavioral therapy (CBT). Observations were performed over an extended period of time (October 2016 – September 2017), which made it possible to repeatedly observe similar situations, and to track the over-all treatment process of several patients. This amounted to 130 h of observations, which were recorded in detailed field notes (Emerson et al., 2011).

### Theoretical Framework

We understand clinical expertise in relation to skills. Skills can be studied with regard to the way in which experts are *situated* (Rietveld, 2008). Experts, on the one hand, are situated in their direct surroundings. When one focuses on particular real-world situations, it is possible to observe at which aspects of their surroundings clinicians are directed and how they interact with it. In the section “Results” we will call this focus on the particular situation *the zoomed-in perspective*.

On the other hand, we assume that experts are situated in a sociocultural practice. In a practice, skills are developed and sustained in relation to others. Given that they share skills, various members of a practice act in more or less regular and stable ways (Rietveld and Kiverstein, 2014). By observing multiple clinicians repeatedly and over an extended period of time, we are in the position to trace the regularities in behavioral patterns that are characteristic for the expert practice of DBS optimization (van Dijk and Rietveld, 2017). In the section “Results” we will call our focus on these regularities *the zoomed-out perspective*.

So, understanding expertise in relation to situatedness makes us not only pay attention to what clinicians say, but also to what they do, to their interaction with particular details of their social and material surroundings, and to regular ways of doing things that are shared by different clinicians (Greenhalgh and Swinglehurst, 2011).

### Analysis

We believe that there is no predefined right or wrong answer to the question how the effect of DBS can best be understood. Instead, we assume, it is more productive to assess the (clinical) relevance of our interpretation of what this effect looks like. This was done by means of theoretical sampling and triangulation, which are important methodological tools to keep an open view and reduce the possibility of bias (Mol, 2002; Corbin and Strauss, 2008). *Theoretical sampling* implies going back and forth between data-acquisition and data-interpretation: hypotheses generated in earlier interviews and observations were tested in subsequent interviews and observations, which made it possible to prove and disprove preliminary interpretations, including those that might be based on preconceived notions of the authors. This resulted in a back-and-forth process in which theoretical insights were gradually developed and sharpened by practice. *Triangulation* implies the cross-checking of different data-sources: interpretations of what a clinician had said during an interview were compared to observations of how this clinician acted in practice. Moreover, we asked clinicians to give feedback both on the presentation of our results in a focus group with them and on preliminary versions of this paper.

## Results

We present our findings by means of a selection of fragments from observations and interviews. These fragments should prove their relevance by giving the reader an understanding of what DBS effects look like from the perspective of an expert clinician.

### Zooming In: A Particular Clinical Situation

Every session, clinicians evaluate the effects of their latest adjustment in stimulation parameters and, consequently, decide whether additional adjustments are required. Below is a description of how such a session usually begins. The patient, Mrs P. returns to the outpatient clinic 2 weeks after her DBS has been switched on.

Mrs P. is seated with her back toward us and the clinician calls her name. She turns around. Contrary to last time, we see a woman with colorful clothing. The clinician extends his hand, whereupon she returns a broad smile. The clinician introduces me [the first author]: “you already know each other, right?” Then, with a gesture, he invites her to walk in front of him to the consulting room. While walking, he asks: “You bought a puppy. How is she?” “How wonderful that you remember that!” Mrs P. replies. The clinician responds by making a small joke. The clinician walks quite fast, with the patient struggling, but succeeding, to keep up. While walking he asks her whether she wants coffee, tea, espresso, or cappuccino. After shaking hands with the junior residents who were waiting in the consulting room, the clinician is seated in front of Mrs P. and reclines. Mrs P. begins by mentioning that she is doing well, upon which the clinician replies: “I can see it.”

We chose to highlight this particular situation because it shows how fast and intuitive the evaluation of DBS effects can be. The example indicates how a skilled clinician rapidly obtains an impression of how the patient is doing. Notably, this happens already before they have arrived at the consulting room. Once there, the clinician will ask the patient systematically how intense her obsessions are and to what extent she can resist performing compulsions. Yet, without having asked any such formal questions, the clinician can already *see* that Mrs P. is doing well, and that, consequently, no additional adjustments in DBS stimulation parameters are necessary.

The clinician’s evaluation that it is going better with the patient is not solely the result of the patient saying so. In various ways he has gathered knowledge on how Mrs P. is doing. That knowledge was not just there, it has been actively obtained. By performing various kinds of actions, we noticed, clinicians elicit certain responses in the patient, which inform them on the effects of DBS. Table 1 gives a (non-exhaustive) list of things clinicians do to assess how the patient is doing.

**TABLE 1:** Diagnostic actions.

Extending a hand
Calling a name
Smiling
Presenting the patient with a choice (for coffee, for various treatment options)
Waiting for the patient to take the initiative
Chit-chatting
Making a joke
Asking questions (about the patient’s dog, about the severity OCD symptoms)
Asking for a clarifying example
Consulting the opinion of family-members
Being alert for a casual remark (for instance about forgetting compulsions)
Rating clinical scales, such as Yale-Brown Obsessive-Compulsive Scale (YBOCS)
Adjusting DBS stimulation parameters
Encouraging the patient to do a small exposure exercise

Which of these listed action possibilities is relevant at a given moment is determined, amongst other things, by the social and material structure of the outpatient clinic. The hallway with the coffee machine, for instance, affords making light conversation, whereas the possibility of asking serious personal questions about the patient’s OCD becomes appropriate only in the privacy of the consulting room. Furthermore, the custom of introducing people to each other in combination with the researcher’s presence at their interaction in the waiting room makes it relevant to the clinician to direct the patient’s attention to the researcher. When the concrete situation in which clinicians’ decision making takes place is taken into account this brings into view that, to some degree, their actions are structured by their social and material surroundings.

Moreover, we observed that clinicians act in response to a dynamically changing situation. As the situation is changed by the clinician’s and patient’s actions, new possibilities for action arise. The clinician’s action of asking the patient about her dog, for example, leads to a response of the patient (“how wonderful that you remember that!”). Her response, in turn, invites the clinician to respond by making a joke, and so on, and so forth.

There is no single clear-cut decision-making moment. Clinical judgment is not confined to the moment in which stimulation parameters are adjusted or when the YBOCS is scored. It already takes place in the long chain of actions preceding the moment in which the clinician says “I can see it.” As the clinician responds to the dynamically changing situation, by asking questions, rating scales, and making jokes, his judgment is gradually sharpened.

Taken together, by zooming in on particular clinical situations we found that evaluating DBS effects is an (a) interactive and (b) context-sensitive process that (c) gradually unfolds over time, and (d) requires integration of different sources of knowledge.

### Zooming Out: Across Multiple Sessions

In this section we take a perspective that stretches across multiple sessions. Every session clinicians make one single adjustment in stimulation parameters. This is repeated until they consider that an “optimal” effect has been established. But what is this “optimal” effect?

Optimal is best understood in contrast to sub-optimal or non-optimal. Below is an interview quote from a clinician who describes how he had been treating a patient with CBT. Before patients are qualified to get DBS the completion of at least one course of CBT is required. A central element in CBT is *exposure with response prevention*, which implies that a patient needs to confront an anxiety-provoking situation without performing compulsions. When sustained and repeated, the patient will learn that the consequences she fears do not occur and that compulsions, therefore, are not necessary.

Below, the clinician describes how he was unable to engage the patient to do the exposure exercises. Her anxiety was simply too strong. He had been “pulling and pushing,” “trying every other technique,” but his attempts failed. He contrasts this with what happened when the patient got DBS and this started working:

“What we asked her to do in therapy, before she had DBS, for at least 100.000 times, she now sees as an achievable option. […] Suddenly she could reflect, could finally say ‘I just did it, I did not perform the compulsions’. There was, as it were, more space in her head to look at her emotional life differently. That was really remarkable. I have seen this woman in therapy before and back then she could not *be moved*. [our italics].”

The clinician who first “pushed and pulled” in vain can now feel that the patient is *being moved*. *Being moved* is a characterization that captures the contrast between a responsive and an unresponsive OCD patient. This characterization indicates a change in the patient’s pattern of responses. Expert clinicians share a sensitivity to this kind of change. They keep optimizing DBS stimulation parameters until they sense that this change in responsiveness has occurred.

The interview quote captures our general finding of how the phenomenon of *being moved* reflects change in three interrelated domains: (1) behavior, (2) emotion, and (3) active engagement. First, there is movement in a literal, behavioral sense. Clinicians observe an “expanding perimeter” of patients’ life worlds as they spend less time and energy on compulsions and stop avoiding places. Patients open up to a broad range of daily life activities, instead of being restricted to a narrow range of compulsive actions.

Second, patients are also moved in an emotional sense. This is illustrated in the case of a patient who, upon looking around after her DBS is switched on, sees her husband in tears and promptly starts to cry herself. Clinicians sense that patients are more easily motivated: the “threshold for engaging in various kinds of activities has been lowered” and “blockages and inhibitions decrease.” Clinicians are emotionally involved in this process themselves. The frustration and dissatisfaction they feel when they push and pull in vain over and over again makes way for enthusiasm and hope as they sense that the patient is being moved.

Third, patients are being moved in the sense that they can be *more actively engaged* in their process of recovery. Clinicians notice that their relationship with the patient becomes more interactive and reciprocal. They are no longer “pulling and pushing” alone and in vain. We observed, for instance, how an OCD-patient who was admitted because she feared harming herself and others suggested she might go home for short periods of time to do little tasks, as watering plants or checking mail, to expose herself gradually to the fear of being at home on her own. More generally, DBS clinicians regard this change in attitude a “window of opportunity”, as the patient’s active participation opens up possibilities for additional psychotherapeutic interventions, such as CBT, which require patients to be actively engaged in their process of recovery.

## Discussion

### Summary of the Findings

We studied how clinicians evaluate DBS effects to understand the process of improvement with DBS in severe OCD patients. We described this process from two perspectives. The first zoomed in on what happens within one treatment session. The second perspective zoomed out across multiple sessions to characterize the change that the different clinicians aim to establish in patients over time. The zoomed in perspective reveals that evaluation of DBS is (a) an interactive and (b) context-sensitive process which (c) gradually unfolds over time and (d) requires integration of various sources of knowledge. The zoomed out perspective reveals that clinicians adjust DBS settings gradually up to the point where the patient is *being moved* with regard to behavior, emotion, and active engagement.

### Implications

Although this study is the first to specifically focus on the perspective of expert clinicians, it is not the first to explore the effects of DBS in OCD. Our research group did phenomenological interviews with 18 OCD patients on the way in which DBS impacts their “lived experience” and field of action possibilities (de Haan et al., 2015, 2017). Other authors, who also use a target in the internal capsule, have interviewed 9 OCD and 6 MDD patients on their experiences of how DBS impacts their actions, decision-making and relationships (Klein et al., 2016). The findings of these studies converge with our finding that, for most patients, the threshold to engage in activities is decreased and a broader range of action possibilities becomes attractive instead of just the narrow range of compulsive routines.

Together with findings from the two interview studies in patients, our study may shed new light on the ethical debate about DBS. It is puzzling so far to know whether and to what extent DBS threatens or supports agency, autonomy, personal identity, and the self. In this debate several authors conceptualize agency as a relational and gradual phenomenon (Baylis, 2013; de Haan et al., 2015, 2017; Goering et al., 2017; Gallagher, 2018). In a relational understanding of agency the DBS device is seen as just one of many aspects that, together, influence the patient’s ability to reach goals, like family members, clinicians, and smartphones do. In a view where many aspects co-determine a person’s agency it makes sense to see the difference in agency of typical people and OCD-patients as a matter of degree. This is also what we found: when DBS starts working the openness to a range of action possibilities gradually increases.

The effects of DBS involve mechanisms of action on different levels of complexity (Jakobs et al., 2019). Our finding of how patients with DBS open up to a broader range of action possibilities resembles the construct of *cognitive flexibility*, which is described as the ability to shift the focus of attention and to acquire new responses in the face of changing environmental demands. Improvements in cognitive flexibility with active DBS have been associated with the activity of distinct brain networks in 5 OCD patients (Tyagi et al., 2019) and with particular patterns in oscillatory activity of frontal neural populations in 2 OCD and 12 MDD patients (Widge et al., 2019).

Given the fact that not only OCD but also MDD patients are included, the above discussed interview and mechanistic studies indicate that the effect we found might be more broadly applicable than to our limited case of OCD alone. Increasingly *being moved* with regard to behavior, emotion, and active engagement may be a general effect of DBS, at least for this capsular site.

#### Relating Perspectives Using Radical Embodied Cognitive Science

We studied the improvement with DBS in OCD patients by interviewing and observing clinicians. In order to show how the perspectives of clinician and patient are integrated, we will now analyze our findings in terms of the theoretical framework of radical embodied cognitive science (RECS) (Thompson, 2007; Chemero, 2009; Noë, 2012; Bruineberg and Rietveld, 2014; McGann, 2014; Gallagher, 2017; Rietveld et al., 2018). Like the relational and gradual view on agency discussed above, RECS takes into account how a person is *situated* within a broader context. More in particular, RECS focuses on the dynamics between an individual and its environment, regarding an individual’s actions as ways of *responding* to the possibilities for action (affordances) the environment offers.^1^

In radical embodied cognitive science terms, patients and clinicians can be seen as parts of each other’s environment. By changing the shared situation in particular ways, as was described in the first part of the section “Results,” clinicians get feedback on the patient’s responsiveness to action possibilities. Somewhat like a wrist is palpated to find out whether it is broken, DBS clinicians systematically probe (Harris, 2015) the OCD patient’s responses. For instance by extending a hand or by asking a question, expert clinicians create possibilities for action for the patient (to shake the hand, to answer the question), putting themselves in a position to assess which action possibilities a patient does and does not respond to.

This integrated understanding of what a patient does and does not respond to is what in RECS terms can be called the patient’s *selective openness* to possibilities for action (Bruineberg and Rietveld, 2014; Rietveld et al., 2018). Selective openness implies that at any given moment some possibilities for action are more attractive to a person than others. It is precisely because clinicians continuously attune to the selective openness of the patient (zoomed in) that they are able to sense when the patient is being moved, that is, when the patient’s selective openness changes (zoomed out).

So, expert clinicians’ sensitivity to when patients are being moved by DBS rests on their ability to attune to the patients’ responses. This ability to attune to OCD patients, we assume, is the result of many interactions with OCD patients in the past. This is knowledge in a form that cannot be straightforwardly translated into words or numbers. It is embodied knowledge. It is knowing how to respond appropriately to a particular patient in a particular situation. This knowledge, as we displayed in this study, can be studied when one zooms in on these particular situations and observes how clinicians interact with patients.

#### DBS as a Holistic Treatment

When OCD patients have reached the point where they are *being moved* they are not there yet. This point marks the beginning of a transition in which patients are more and more engaged in their process of improvement. As the patient is being moved, new action possibilities emerge, both for the patient and the clinician. One of these possibilities is CBT, in which remaining symptoms are reduced and healthy behavioral repertoires are expanded. Engaging in CBT is crucial for recovery. If one stays at home, without engaging in new action possibilities, clinicians observed, old habits will eventually return. The movement which was initiated by DBS needs to be kept going.

The importance of actively engaging in new action possibilities, of seeing *being moved* as the beginning of a transition, shows how DBS and CBT are mutually reinforcing treatments. DBS creates the conditions for CBT and CBT, in turn, increases DBS efficacy (Mantione et al., 2014) and prevents relapse. The relation between DBS and CBT also shows how, from the perspective of expert clinicians, diagnosis, and treatment are interrelated. Already during the optimization of DBS stimulation parameters, clinicians start with small CBT exercises. When these exercises are successful this is *therapeutic* in the sense that a patient learns that what she fears does not occur. At the same time the CBT exercise is also *diagnostic*: it informs the clinician whether the stimulation parameters of the DBS are effective or not.

The way in which expert clinicians integrate DBS and CBT and diagnosis and treatment stresses the importance of seeing DBS treatment as holistic. Holism, from a RECS-perspective, means that a patient (or brain) is not regarded in isolation, but as part of a larger brain-person-environment system. This holism is reflected in the skills of expert clinicians, who are open to a range of action possibilities offered by the different aspects of this brain-person-environment system. This includes the possibility of adjusting DBS stimulation parameters (thereby affecting the patient’s brain) as well as the possibility of, say, taking the patient outdoors for an exposure exercise (thereby changing the patient’s environment). Both interventions have the potential to affect how the patient relates to his or her environment.

### Limitations

A limitation of this study is that its findings cannot be expressed in quantitative terms. Our qualitative work can be seen as complementary to studies that use quantitative means of evaluating OCD improvement, such as the YBOCS (Denys et al., 2010). Moreover, explorative studies like ours can open up new directions for quantitative enquiry. Based on our findings one might, for example, operationalize the clinicians’ strategy of evaluating DBS effects in terms of changes in the way patients relate to their environment. Simple tools like GPS-trackers could, for instance, quantify the expanding perimeter of a DBS-patient’s activities in the living environment as his or her openness to the world increases.

A further limitation is that the authors, as a result of their professional and academic backgrounds, have expectations that might have biased data-interpretation. Having published on RECS before, the authors have a tendency to interpret their current findings in light of this past work. Additionally, the first and last author are clinicians themselves, which could have limited their ability to ask “naïve” question that might probe clinicians to explain basic aspects of their practice. We have tried to minimize the possibility of bias by means of theoretical sampling and triangulation. Moreover, we believe that our particular backgrounds and related attitudes are important as they shape our sensitivity to aspects of the practice of DBS optimization that remain often out of view (Mol, 2002, 2008).

This study foregrounded the perspective of clinicians. This, however, does not mean that we see patients as mere passive subjects. What’s more, their active participation in the treatment is crucial to its success. Our research shows that clinicians are directed at creating conditions for the patient to become actively involved in his or her process of improvement. Furthermore, in earlier work our research group described DBS-induced changes in the phenomenology of OCD patients who are comparable to the patients figuring in the present study (de Haan et al., 2013, 2015, 2017). In contrast to this former study, we focused primarily on the clinicians and the activities they perform to assess and establish these DBS-induced changes. Via a different route, our study on clinicians nevertheless arrived at the same conclusion: in both studies the effect of DBS has been characterized as a restructuring of patients’ openness to the world.

The emphasis we put on clinicians’ responsiveness might make it seem like everything they do always goes smoothly, almost effortlessly. However, note that we also described how clinicians were initially “pulling and pushing” a severely ill patient without success. Only for reasons of clarity, we chose not to include more descriptions of episodes in which attempts to improve a situation failed. For the same reason, we did not include situations in which clinicians were in doubt or were engaged in multidisciplinary discussions^2^, as happened with patients that did not respond to DBS, that suffered from intolerable side effects or whose comorbid disorders interfered with the treatment.

Finally, it is not clear to what extent our findings apply to other DBS centers, where things are organized differently (e.g., not all centers add CBT to DBS, not all centers implant the electrode in the vALIC), or to other OCD treatments, and DBS in other illnesses, such as Major Depressive Disorder and Parkinson’s Disease. Nevertheless, we believe that the importance of attuning to a patient is something many clinicians will recognize. This study has tried to make a case for why clinical expertise is so important to effective treatment.

## Conclusion

Obsessive-compulsive disorder patients’ improvement with DBS can be understood as a change in selective openness and responsiveness to their environment. The threshold for patients to engage in activities is decreased and a broader range of action possibilities becomes attractive to the patient instead of just the narrow range of compulsive routines. Clinical expertise enables clinicians to attune to the patient’s responses and, thereby, to sense this DBS-induced change. From that moment on, patients can be actively engaged to further expand their openness to the world by means of additional psychotherapeutic interventions, such as CBT.

## Data Availability Statement

The datasets generated for this study are available on request to the corresponding author.

## Ethics Statement

Ethics approval was not required as per applicable institutional and national guidelines and regulations. Based on the Research Involving Human Subjects Act (Wet Medisch-Wetenschappelijk Onderzoek met Mensen), the Medical Ethics Review Committee of the Academic Medical Centre (AMC), Meibergdreef 9 in Amsterdam, Netherlands, declared that no official approval was required as our study did not subject persons to interventions or behavioral regulations. The clinicians, who were the primary participants of this study, provided verbal informed consent to being interviewed and observed and were offered the opportunity to read and comment on this manuscript. Sessions where patients were present were only attended when the patient provided verbal informed consent to the researcher’s attendance and anonymized field note taking. Written informed consent was obtained from Mrs P. (not her real initials) for the publication of the case description in this article, including all identifiable information.

## Author Contributions

MW and ER performed the interviews. MW did participant observation. MW analyzed the data. MW wrote the first draft of the manuscript. All authors contributed to the conception, design of the study, manuscript revision, and read and approved the submitted version.

## Conflict of Interest

The authors declare that the research was conducted in the absence of any commercial or financial relationships that could be construed as a potential conflict of interest.

## Footnotes

1 The technical term for possibilities for action is *affordances*, which was first introduced by Gibson (1979). In earlier work we have defined affordances as relations between aspects of the sociomaterial environment in flux and abilities available in an ecological niche or practice (Rietveld and Kiverstein, 2014; Rietveld et al., 2018).

2 Nevertheless, based on our earlier work we think that reflection fits in with the RECS-approach we described: concrete socio-material situations include things other people say and offer possibilities for reflection as well (Rietveld and Kiverstein, 2014; Rietveld et al., 2018). We aim to discuss this in relation to our observations in future work.
